# Clonal evolution characteristics and reduced dimension prognostic model for non-metastatic metachronous bilateral breast cancer

**DOI:** 10.3389/fonc.2022.963884

**Published:** 2022-09-29

**Authors:** Lingyu Li, Jiaxuan Li, Jiwei Jia, Hua He, Mingyang Li, Xu Yan, Qing Yu, Hanfei Guo, Hong Wang, Zheng Lv, Haishuang Sun, Guidong Liao, Jiuwei Cui

**Affiliations:** ^1^ Cancer Center, The First Hospital of Jilin University, Changchun, China; ^2^ School of Mathematics, Jilin University, Changchun, China; ^3^ National Applied Mathematical Center (Jilin), Changchun, China; ^4^ College of Electronic Science and Engineering, Jilin University, Changchun, China; ^5^ Department of Translational Medicine, Geneplus-Beijing, Beijing, China

**Keywords:** metachronous bilateral breast cancer, SEER database, clonal evolution, nomogram, prognostic evaluation

## Abstract

**Background:**

How to evaluate the prognosis and develop overall treatment strategies of metachronous bilateral breast cancer (MBBC) remains confused in clinical. Here, we investigated the correlation between clonal evolution and clinical characteristics of MBBC; we aim to establish a novel prognostic model in these patients.

**Methods:**

The data from Surveillance, Epidemiology, and End Results (SEER) database and the First Hospital of Jilin University were analyzed for breast cancer–specific cumulative mortality (BCCM) by competing risk model. Meanwhile, whole-exome sequencing was applied for 10 lesions acquired at spatial–temporal distinct regions of five patients from our own hospital to reconstruct clonal evolutionary characteristics of MBBC. Then, dimensional-reduction (DR) cumulative incidence function (CIF) curves of MBBC features were established on different point in diagnostic interval time, to build a novel DR nomogram.

**Results:**

Significant heterogeneity in genome and clinical features of MBBC was widespread. The mutational diversity of contralateral BC (CBC) was significantly higher than that in primary BC (PBC), and the most effective prognostic MATH ratio was significantly correlated with interval time (*R* ^2^ = 0.85, *p<* 0.05). In SEER cohort study (*n* = 13,304), the interval time was not only significantly affected the BCCM by multivariate analysis (*p*< 0.000) but determined the weight of clinical features (T/N stage, grade and ER status) on PBC and CBC in prognostic evaluation. Thus, clinical parameters after DR based on interval time were incorporated into the nomogram for prognostic predicting BCCM. Concordance index was 0.773 (95% CI, 0.769–0.776) in training cohort (*n* = 8,869), and 0.819 (95% CI, 0.813–0.826) in validation cohort (*n* = 4,435).

**Conclusions:**

Bilateral heterogeneous characteristics and interval time were determinant prognostic factors of MBBC. The DR prognostic nomogram may help clinicians in prognostic evaluation and decision making.

## Introduction

Metachronous bilateral breast cancer (MBBC) with high heterogeneity accounts for 3% of total breast cancer (BC) (1, 2). Owing to the increasing morbidity of BC, prolongation of survival time, and improvement of detection rate, a growing number of patients with BC are diagnosed as contralateral disease and treated with mastectomy (2–4). Recently, majority of studies focused on the risk factors for the formation of contralateral breast cancer (CBC) in patients with primary breast cancer (PBC) (5) and expected to prevent the occurrence of CBC *via* bilateral mastectomy (6, 7), whereas, for clinical practice, it is not yet clear whether patients would benefit from bilateral mastectomy in terms of mortality (6). More importantly, once diagnosed as CBC, how to develop overall treatment strategies and evaluate the prognosis of these MBBC remains confused in clinical (8–11). Actually, most clinical understanding of MBBC is obviously distinct from unilateral breast cancer (UBC) (12, 13). For a patient with MBBC, clinical and pathological characteristics between the PBC and CBC can be consistent or inconsistent, which makes the subtypes of a characteristic multiplying (**Table 1**). Applying the prognosis evaluation system of UBC to MBBC will be complicated and inapplicable; thus, it is urgently needed to build an evaluation model for predicting the prognosis of MBBC.

**Table 1:** Competing risk model for MBBC.

Variable	No. of	%	Univariate analysis				Multivariate analysis			
	patients		*P-*value	Sub-distri-bution HR	95% CI low	95% CI upp	*P*-value	Sub-distribution HR	95% CI low	95% CI upp
**Age at diagnosis of CBC (years)**										
<= 40	411	3.09	Ref				Ref			
41-50	1493	11.22	< 0.001	0.66	0.55	0.80	0.229	0.76	0.49	1.19
51-60	2739	20.59	< 0.001	0.49	0.41	0.58	0.688	0.92	0.60	1.40
> 60	8661	65.10	< 0.001	0.36	0.30	0.42	0.738	0.93	0.61	1.42
**Interval time** (**years**)										
Continuous Variable form	13304		< 0.001	0.93	0.92	0.94	**< 0.001**	**0.92**	**0.89**	**0.95**
<= 7	7551	56.76	Ref							
> 7	5753	43.24	< 0.001	0.56	0.51	0.61				
**Race**										
White, non-Hispanic	10731	80.68	Ref				Ref			
Black, non-Hispanic	1308	9.83	< 0.001	1.63	1.45	1.84	0.383	0.88	0.67	1.17
Other, mixed	1261	9.48	0.542	0.95	0.82	1.11	0.198	1.22	0.90	1.65
Marital status										
Non-P/Non-P	4371	35.20	Ref				Ref			
Non-P/With-P	382	3.08	0.489	0.91	0.71	1.18	0.732	0.91	0.55	1.53
With-P/Non-P	1304	10.50	0.017	0.83	0.71	0.97	0.240	0.78	0.52	1.18
With-P/With-P	6359	51.22	0.004	0.88	0.80	0.96	0.181	0.88	0.72	1.06
**T stage**										
T1/T1	6069	51.55	Ref				Ref			
T1/T2	1438	12.22	< 0.001	2.41	2.09	2.77	**< 0.001**	**2.23**	**1.67**	**2.99**
T1/T3-T4	285	2.42	< 0.001	6.03	4.89	7.43	**< 0.001**	**4.70**	**3.01**	**7.35**
T2/T1	2169	18.43	< 0.001	1.92	1.68	2.19	**0.001**	**1.65**	**1.24**	**2.20**
T2/T2	757	6.43	< 0.001	3.40	2.89	3.99	**< 0.001**	**3.06**	**2.23**	**4.19**
T2/T3-T4	237	2.01	< 0.001	8.98	7.16	11.25	**< 0.001**	**5.19**	**2.99**	**8.99**
T3-T4/TI	437	3.71	< 0.001	3.23	2.63	3.96	**0.001**	**2.28**	**1.42**	**3.67**
T3-T4/T2	197	1.67	< 0.001	6.55	5.12	8.38	**< 0.001**	**3.30**	**2.03**	**5.37**
T3-T4/T3-T4	183	1.55	< 0.001	15.24	12.21	19.02	**< 0.001**	**4.25**	**2.38**	**7.57**
**N stage**										
N0/N0	6740	57.22	Ref				Ref			
N0/N1	1236	10.49	< 0.001	2.28	1.96	2.65	**<.001**	**1.76**	**1.29**	**2.40**
N0/N2-N3	499	4.24	< 0.001	5.74	4.88	6.75	**<.001**	**3.60**	**2.49**	**5.21**
N1/N0	1620	13.75	< 0.001	1.73	1.49	2.01	**0.006**	**1.51**	**1.13**	**2.03**
N1/N1	404	3.43	< 0.001	4.00	3.28	4.87	**<.001**	**3.46**	**2.42**	**4.94**
N1/N2-N3	230	1.95	< 0.001	9.41	7.75	11.43	**<.001**	**4.58**	**3.07**	**6.84**
N2-N3/N0	615	5.22	< 0.001	3.59	3.03	4.25	**<.001**	**3.24**	**2.24**	**4.68**
N2-N3/N1	194	1.65	< 0.001	6.29	4.97	7.97	**<.001**	**4.10**	**2.45**	**6.87**
N2-N3/N2-N3	241	2.05	< 0.001	14.51	12.09	17.41	**<.001**	**6.14**	**3.82**	**9.88**
**Tumor grade**										
I-II/I-II	4738	45.58	Ref				Ref			
I-II/III-IV	1562	15.03	< 0.001	2.05	1.78	2.35	**0.032**	**1.34**	**1.02**	**1.76**
III-IV/I-II	1998	19.22	< 0.001	1.32	1.14	1.52	0.338	1.15	0.86	1.55
III-IV/III-IV	2098	20.18	< 0.001	2.80	2.49	3.15	**0.001**	**1.63**	**1.23**	**2.16**
**Pathological type**										
IDC/IDC	6841	51.42	Ref				Ref			
IDC/ILC	825	6.20	0.727	0.97	0.81	1.16	0.813	0.95	0.61	1.48
IDC/Other	1686	12.67	0.384	1.06	0.93	1.20	0.356	1.15	0.86	1.54
ILC/IDC	536	4.03	0.477	0.93	0.75	1.15	0.860	1.05	0.60	1.86
ILC/ILC	278	2.09	< 0.001	1.74	1.38	2.19	0.220	1.41	0.81	2.45
ILC/Other	177	1.33	0.133	1.26	0.93	1.70	0.717	1.13	0.59	2.15
Other/IDC	1909	14.35	0.731	1.02	0.91	1.15	**0.011**	**1.40**	**1.08**	**1.81**
Other/ILC	313	2.35	0.785	1.04	0.79	1.38	0.257	1.35	0.81	2.25
Other/Other	739	5.55	0.158	1.13	0.95	1.35	0.187	1.30	0.88	1.90
**Surgery method**										
BCM/BCM	2699	42.24	Ref				Ref			
BCM/SM	814	12.74	0.520	1.09	0.83	1.43	0.630	0.92	0.66	1.28
BCM/RM	541	8.47	< 0.001	2.25	1.80	2.83	0.708	0.94	0.69	1.29
SM/BCM	124	1.94	0.073	1.59	0.96	2.65	0.902	0.95	0.45	2.03
SM/SM	332	5.20	0.353	0.81	0.52	1.26	**0.015**	**0.48**	**0.27**	**0.87**
SM/RM	154	2.41	<.001	2.41	1.67	3.49	0.922	0.98	0.60	1.60
RM/BCM	330	5.17	<.001	2.09	1.56	2.78	0.685	0.92	0.60	1.41
RM/SM	619	9.69	<.001	1.59	1.23	2.06	0.580	0.91	0.64	1.28
RM/RM	776	12.15	<.001	2.87	2.38	3.46	0.456	0.90	0.68	1.19
**ER status**										
+/+	6286	61.02	Ref				Ref			
+/-	1279	12.42	<.001	1.73	1.51	1.98	0.060	1.29	0.99	1.68
-/+	1480	14.37	0.685	0.97	0.83	1.13	**0.006**	**0.64**	**0.46**	**0.88**
-/-	1256	12.19	<.001	2.19	1.93	2.50	0.103	1.28	0.95	1.72
**PR status**										
+/+	4290	43.49	Ref							
+/-	2213	22.44	<.001	1.69	1.49	1.91				
-/+	1592	16.14	0.738	0.97	0.82	1.15				
-/-	1769	17.93	<.001	2.07	1.82	2.36				
**HER2 status**										
+/+	23	4.83	Ref							
+/-	26	5.46	<.001	0.00	0.00	0.00				
-/+	54	11.34	0.309	0.38	0.06	2.42				
-/-	373	78.36	0.454	0.57	0.13	2.46				

MBBC, metachronous bilateral breast cancer; No., number; sub-distribution HR, subdistribution hazard ratio; CI, confidence interval; low, lower bound of confidence interval; upp, upper bound of confidence interval; CBC, contralateral breast cancer; Ref, reference; Non-P, without partner at diagnosis (single, divorced, widowed, or separated); With-P, with partner at diagnosis (married, unmarried or domestic partner, or same sex or opposite sex partner); T, tumor; N, node; IDC, invasive ductal carcinoma; ILC, invasive lobular carcinoma; BCS, breast-conserving surgery; SM, simple mastectomy; RM, radical mastectomy; ER, estrogen receptor; PR, progesterone receptor; HER2, human epidermal growth factor receptor 2; +, positive; -, negative. The meaning of symbol “<=” was “less than or equal to”. The meaning of symbol “<” was “less than”. The meaning of symbol “>” was “more than”. The meaning of the bold values means these p values were less than 0.05 and considered as having statistical significance.

Considering the lower morbidity of MBBC than UBC, we collected and analyzed the clinicopathologic and prognostic data from Surveillance, Epidemiology, and End Results (SEER) database. Each patient with MBBC in SEER database had two recordings for PBC and CBC, respectively, but the follow-up outcome was the same. Clinicopathologic characteristics were reclassified into new variables to unify the recordings to represent one patient for the further research (**Table 1**). In view of the relatively long overall survival of BC, to exclude the death form non-cancer specific causes, competing risk modeling was used to select the independent risk factors that affected the follow-up outcomes (with *p*< 0.05 after multivariate analysis in **Table 1**). Although this classification method combined the PBC and CBC to evaluate the prognosis, the multifarious subtypes of each variable limited the clinical application.

The concordance of molecular subtype was closely associated with survival outcome in synchronous BBC and MBBC. Among them, patients with MBBC had lower molecular subtype concordance rate than patients with synchronous BBC (14). The spatial–temporal heterogeneity (15) between PBC and CBC, in terms of the clinical, molecular, and genomic characteristics (16), makes it more complicated and confused to fully understand this disease. In this study, we investigated the regularity of heterogeneity distribution and clonal evolution characteristics between PBC and CBC and firstly found that the interval time dimension was a determinant prognostic factor of MBBC. Then, with the help of mathematical model, we reclassified the meaningful variables form multivariate analysis of competing risk modeling (**Table 1**) depended on interval time, which reduced the number of subtypes efficiently and was named as dimensional reduction (DR) (**Table 2**). Based on the novel DR competing risk model (**Table S5**), which reanalyzed the DR variables, a concise and precise DR nomogram was established to help clinicians in clinical prognostic evaluation and decision making.

**Table 2:** Prognostic score assignment and DR algorithm.

Variable	Score of PBC	Score of CBC	Total score(range)	Cutoff value of total score	DR stage
**T stage**			(1.00–3.00)		**T^DR^ stage**
T1/T1	1	1	=1*Wpt+1*Wct	1.00~1.50	1
T1/T2	1	2	=1*Wpt+2*Wct	1.51~2.00	2
T1/T3-T4	1	3	=1*Wpt+3*Wct	2.01~2.50	3
T2/T1	2	1	=2*Wpt+1*Wct	2.51~3.00	4
T2/T2	2	2	=2*Wpt+2*Wct		
T2/T3-T4	2	3	=2*Wpt+3*Wct		
T3-T4/TI	3	1	=3*Wpt+1*Wct		
T3-T4/T2	3	2	=3*Wpt+2*Wct		
T3-T4/T3-T4	3	3	=3*Wpt+3*Wct		
**N stage**			(1.00–3.00)		**N^DR^ stage**
N1/N1	1	1	=1*Wpn+1*Wcn	1.00~1.50	1
N1/N2	1	2	=1*Wpn+2*Wcn	1.51~2.00	2
N1/N3-N4	1	3	=1*Wpn+3*Wcn	2.01~2.50	3
N2/N1	2	1	=2*Wpn+1*Wcn	2.51~3.00	4
N2/N2	2	2	=2*Wpn+2*Wcn		
N2/N3-N4	2	3	=2*Wpn+3*Wcn		
N3/N1	3	1	=3*Wpn+1*Wcn		
N3/N2	3	2	=3*Wpn+2*Wcn		
N3/N3-N4	3	3	=3*Wpn+3*Wcn		
**Grade**			(1.00~2.00)		**Grade^DR^**
I-II/I-II	1	1	=1*Wpg+1*Wcg	1.00~1.50	1
I-II/III-IV	1	2	=1*Wpg+2*Wcg	1.51~2.00	2
III-IV/I-II	2	1	=2*Wpg+1*Wcg		
III-IV/III-IV	2	2	=2*Wpg+2*Wcg		
**ER status**			(1.00~2.00)		**ER^DR^ status**
+/+	1	1	=1*Wpe+1*Wce	1.00~1.50	1
+/-	1	2	=1*Wpe+2*Wce	1.51~2.00	2
-/+	2	1	=2*Wpe+1*Wce		
-/-	2	2	=2*Wpe+2*Wce		

DR, dimension reduction; Wpt, weight of PBC’s T stage for prognostic prediction; Wct, weight of CBC’s T stage for prognostic prediction; Wpn, weight of PBC’s N stage for prognostic prediction; Wct, weight of CBC’s N stage for prognostic prediction; Wpg, weight of PBC’s grade for prognostic prediction; Wcg, weight of CBC’s grade for prognostic prediction; Wpe, weight of PBC’s ER status for prognostic prediction; Wce, weight of CBC’s ER status for prognostic prediction.

## Materials and methods

### Study population

We obtained the study participants from the population-based SEER database (1990–2015) and the First Hospital of Jilin University (2001–2019). With a focus on evaluation of MBBC, we defined survivors of CBC as patients with BBC who survived more than 6 months after the diagnosis of PBC (6). Whereas, fulfilling any one of the following criteria would be excluded: (1) had distant metastases at diagnosis of the primary lesion, (2) less than 18 years of age or older than 97 years of age at diagnosis with PBC, and (3) the duration of follow-up was less than 3 months or withdraw. Finally, we identified 13,304 patients who diagnosed with MBBC between 1990 and 2015 from SEER. Since human epidermal growth factor receptor 2 (HER2) status was unavailable in SEER before 2010, only 476 patients had recordings of HER2 status. The analysis, mathematical operation, and the build of competing risk model and nomogram (training and validation cohort) were mainly based on the data from SEER. Furthermore, we browsed 25,119 patients with BC from the First Hospital of Jilin University range from 2001 to 2019; 89 patients with BBC were included in our study to establish a tentative external validation. Here, we included patients without distant metastases at first diagnosis to minimize the risk of misclassified metastatic disease.

### Whole-exome sequencing and data analysis

We surgically removed sample acquired at spatial–temporal distinct regions from five patients who received chemotherapy. DNA libraries for WGS were generated by Illumina TruSeq DNA Library Preparation Kit (Illumina, San Diego, CA) from shear DNA fragments with a peak of 250 bps, which extracted from tumor tissues (the QIAamp DNA FFPE Tissue Kit, Qiagen, Hilden, Germany). NimbleGen EZ 64M human exome array probes (SeqCap EZ Human Exome Library v3.0) were used in hybridization. DNA sequencing was performed using an HiSeq 3000 instrument (Illumina, San Diego, CA) with 2 × 75 bp paired-end sequencing strategy. Process of reads alignment and calling for somatic single-nucleotide variations (SNVs) are described in the Supplementary Data.

### Dimensional-reduction mathematical model

After plotting the cumulative incidence function (CIF) curves of T stage, N stage, grade, and ER status at different point in interval time by univariate competing risk analysis, the area under the curve of each subgroup was obtained. According to the difference of the areas among subgroups of the variable, the weight used to evaluate the different proportion of PBC and CBC in predicting the cancer-specific death was calculated at a point in time (17, 18). A reference index of the weight of primary or contralateral cancer was normalized and standardized as the reference weight value of each point in interval time (19). To fit the weight values, the nonlinear fitting function with parameters was set and the regression coefficients were worked out.

### Statistical analysis

Competing risk modeling: Follow-up was begun from the diagnosis of CBC to the date of death or the last recording from SEER or the hospital. In SEER cohort, there were 2,357 patients died from cancer and 2,624 patients died for other causes within 25 years of follow-up, which was suitable for breast cancer–specific cumulative mortality (BCCM) calculated by Fine and Gray’s competing risk model (9) to remove interference from other causes of death. We did not censored follow-up at age more than 70 years since other cause deaths could be excluded by the risk-competitive model.

Construction of the nomogram: According to the competing risk model, four independent prognostic variables were included and revised by DR mathematical model. We further screened for prognosis impact factors by Fine and Gray's competing risk regression analysis and constructed a corresponding competing risk nomogram. The eligible patients from SEER were divided into two groups randomly by 2:1: training cohort (*n* = 8,869) and validation cohort (*n* = 4,435). There were no differences in clinical features between the training cohort and validation cohort except in surgery method (**Table S3**). Concordance index (C-index) values were used to measure the discrimination performance and calibration curves were assessed graphically by plotting the observed rates against the nomogram-predicted probabilities *via* a bootstrap method with 1,000 resamples.

Statistics of clinical characteristics between PBC and CBC were analyzed by χ^2^ test used to compare categorical characteristics. In the competing risk model, the clinicopathologic factors affecting the follow-up outcomes independently were selected, subdistribution hazard ratios (SHRs) with 95% confidence interval (CI) were calculated, and CIF curves were plotting using STATA Version 15.0. Other data analyses were performed using R software version 3.6.1 (R Foundation for Statistical Computing). Two-sided *p*< 0.05 was considered statistically significant.

## Results

### Clinical characteristics distribution of MBBC

**Figure 1:**
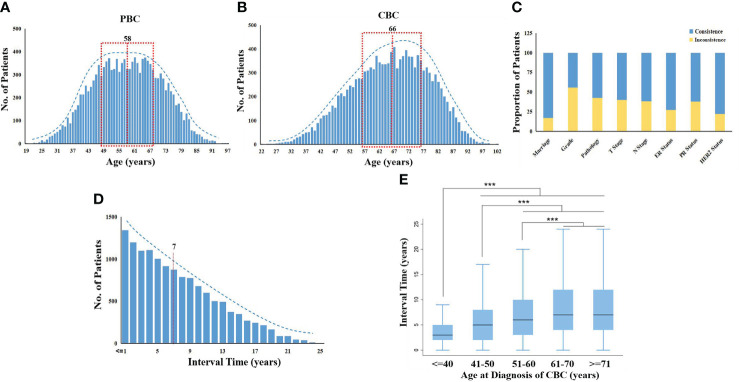
Distribution of clinical characters in MBBC from SEER. **(A, B)** Age distribution in MBBC: the number of patients plotted on the *y*-axis against age on the *x*-axis for PBC **(A)** and CBC **(B)**. The mean age of diagnosis with PBC and CBC was 58 *versus* 66 years, respectively. **(C)** The consistent radio by different clinical features on the *y*-axis against gender on the *x*-axis. **(D)** The number of patients with CBC plotted on the y-axis against the interval time, and the mean was 7 years. **(E)** Patients were divided into five groups according to age at diagnosis of CBC (≤40, 41–50, 51–60, 61–70, ≥71), the range of interval time was counted on the *x*-axis. ***p < 0.001.

Among the 473,909 patients with BC in SEER, 13,304 individuals of MBBC were included, which is consistent with a 3%–4% overall morbidity of MBBC (baseline characteristics in **Tables S1–S2**). Diagnosis of PBC had a peak at approximately age ranged from 48 to 68 years, and incidence of most CBC was at the ages 56 to 76 years (**Figures 1A, B** **)**. The mean age of diagnosis with PBC and CBC was 58 *versus* 66 years, and the interval time between PBC and CBC ranged from 6 months to 25 years (mean interval, 7 years), and the occurrence risk of CBC gradually decreased as the time interval lengthens (**Figure 1D**). In addition, the younger the onset age of CBC means the shorter spacing interval of MBBC that the median interval in patients younger than 40 years was only 3 years (*p<* 0.0001, **Figure 1E**).

All of the clinical characteristics between PBC and CBC were significantly different, including marriage status, differentiation grade, pathology, tumor size, lymph nodes metastasis (LNM), estrogen receptor (ER) status, progestrone receptor (PR) status, and HER2 status (*p<* 0.0001; **Table S2**). Heterogeneity of MBBC was obvious in clinical features, inconsistent proportions between PBC and CBC among the above characteristics were 17.14, 55.77, 42.64, 40.11, 38.18, 27.50, 38.08, and 22.02%, respectively (**Figure 1C**).

### Heterogeneity of somatic mutations and clonal evolution in BBC

**Figure 2:**
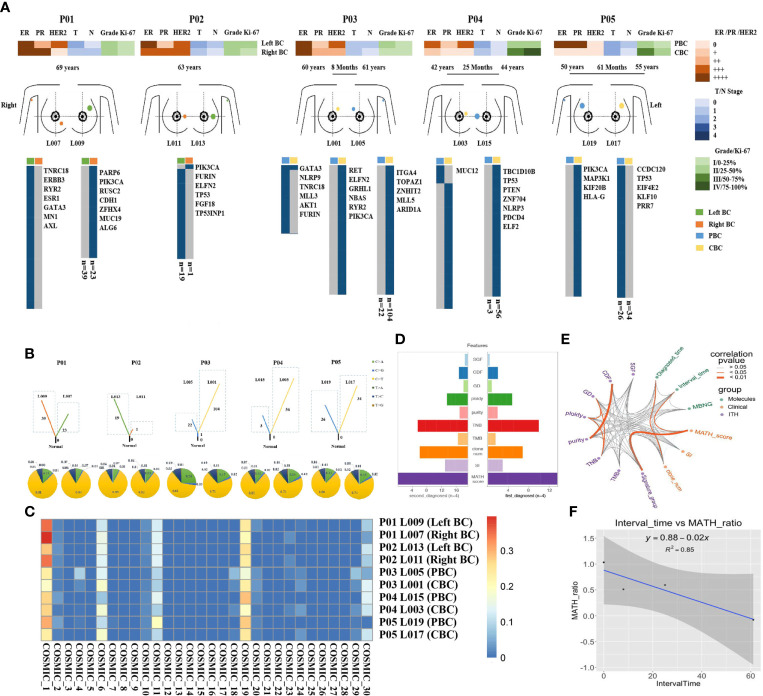
Heterogeneity of somatic mutations and clonal evolution in BBC. **(A)** Heat maps show the clinical characters and individual somatic mutations of five BBC patients with different interval time (rang from less than 6 months to 61 months) in right breast (orange) and left breast (green) of SBBC (P01 and P02), or PBC (blue) and CBC (yellow) of MBBC. The presence (blue) or absence (gray) of each mutation is indicated for every tumor region. Clonal evolution of 10 pathological specimens after operation (L001 to L019) from different spatial regions. **(B)** Fraction of early mutations (trunk) and late mutations (branch) accounted for by each of the six mutation types in all samples. Driver mutations occurring in an APOBEC signature (C > T and C > G mutations) are highlighted with blue and yellow box. **(C)** Heat maps show the common mutational signatures *via* COSMIC. **(D)** The total importance for each feature group. SGF, sub-clonal genome fraction; CDF, cancer DNA fraction; GD, genome doublings; TNB, tumor neoantigen burden; TMB, tumor mutational burden; clone_num, clone numbers; SI, Shannon index. **(E)** The correlation and prognosis importance for 14 features, including clinical (green points), molecular (purple points) characters, and ITH (orange points) was shown by wires. Dark orange wires meant the relevance of each point had statistically significant (*p<* 0.05) and gray wires meant insignificance (*p* > 0.05). **(F)** The relationship of the interval time and MATH-score-ratio was described by regression equation, *y* = 0.88–0.02x.

To evaluate the heterogeneity of nonsilent mutations between bilateral tumor lesion, we sequenced 10 spatially distinct regions from five operable patients with BBC. In terms of a single patient, each mutation defined as ubiquitous (present in bilateral tumor regions) or heterogeneous (present in one side of the lesion). Spatial heterogeneity was identified in all five BBCs, with almost all heterogeneous mutations between bilateral tumor lesion (range: 95.4–100%), except for only one ubiquitous mutation GATA3 in patient P03 (**Figure 2A**).

To further explore the dynamics of the mutational processes shaping BBC genomes over time, the spectra of point mutations in each lesion were dissected. Compared with synchronous BBC (patients P01 and P02), heterogeneous distribution of somatic mutations in MBBC was significantly associated with the sequence of onset and interval time. The mutational diversity of CBC was significantly higher than that of PBC, and the shorter the interval exhibited an increase in somatic mutation of CBC, indicating the poorer prognosis (patients P03–P05, **Figure 2B**).To characterize the genomic instability process between the occurrence of PBC and CBC, we investigated common mutational signatures *via* catalogue of somatic mutation in cancer [COSMIC (20), https://cancer.sanger.ac.uk/cosmic/signatures_v2], which contained signatures 1, 6, 11, and 19. Thereinto, signatures 1 and 11 were closely associated with age of cancer diagnosis and chemotherapy drugs, such as alkylating agent (**Figure 2C**).

To further predict the clinical outcomes of MBBC, we integrated clinical, molecular, and ITH (21) features (measured as the percentage of late mutations (22), highlighted the complex interaction between driver status and tumor heterogeneity (23) from multiple layers, and discovered that several highly correlated features were found to stratify patient outcomes, such as MATH (24) ratio and clone numbers (**Figure S1B**). Plotting the correlation structure across these features, we discovered that clinical feature interval time was significantly associated with MATH ratio (p< 0.05, R2 = 0.85, **Figures 2D–F**), suggesting that time interval between BBC is an important reflection of tumor evolution and clinical prognosis. Furthermore, previous large-scale sequencing of pan-cancer studies had reported that patient outcome could be better predicted by clinical features than by genomic features (25). Thus, a prognostic model based on essential clinical features might stratify the prognosis of MBBC.

### Comprehensive analysis of PBC and CBC in competing risk model

In our study, to identify the essential features, several clinical parameters were included into competing risk models (1): race, the age at diagnose time of CBC, the interval time ranged from PBC to CBC (2); differences of marriage status, tumor size, LNM, grade, pathology, molecular status, and surgery types in PBC and CBC. In univariate analysis of BCCM, almost all of the clinical parameters had significant differences (*p*< 0.000) but for race, marital status, and HER2 status. According to multivariate analysis, interval time (*p*< 0.000), tumor size (*p*< 0.001), LNM (*p*< 0.006), grade (*p<* 0.032), and ER status (*p* = 0.006) between PBC and CBC significantly affected the prognosis of these patients (**Table 1** and **Table S4**). The surgery method (*p >* 0.050) was not a critical factor in prediction of bilateral BCCM.

### Interval time determines the weight of MBBC characteristics in BCCM

**Figure 3:**
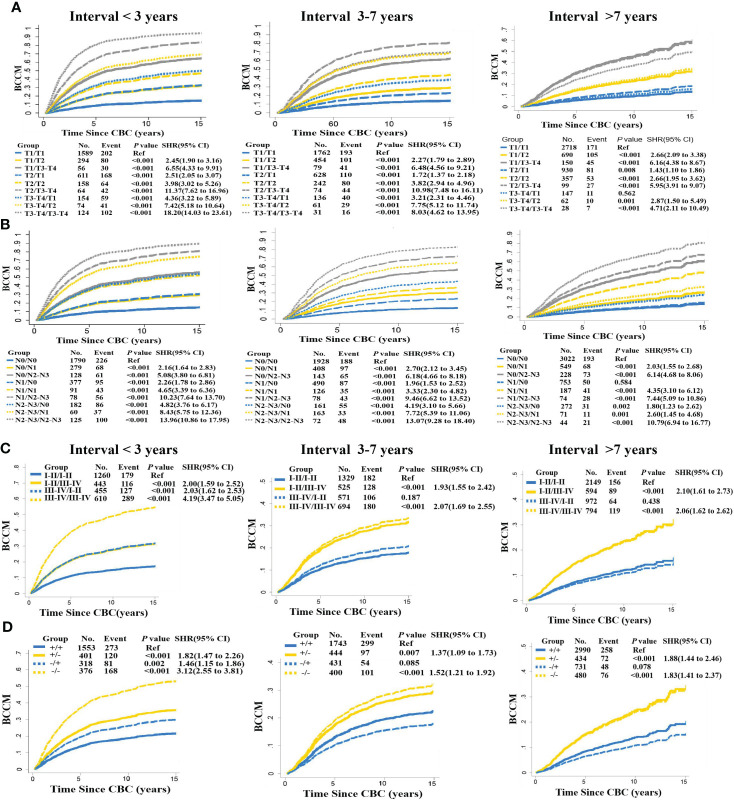
Interval time in stratified BCCM of MBBC characteristics. Risk group stratification within each prognostic factor with distant interval time (< 3, 3–7, > 7 years), including T stage **(A)**, N stage **(B)**, grade **(C)**, and ER status **(D)**. The same features of PBC are reflected with the same line type (solid or dashed line), and the same features of CBC are reflected with the same line color (blue, yellow, or gray).

In view of the importance of interval time in tumor clonal evolution, we conducted further stratified analysis at different intervals based on the above prognostic factors. Estimates for BCCM differed across the interval time, significantly survival discrepancy for MBBC patients with spacing interval< 3 years, 3–7 years and > 7 years. When patients diagnosed with CBC within 3 years, critical clinical features (T stage, N stage, grade, and ER status) of PBC and CBC almost simultaneously inflected the BCCM of patients. Once patients with interval time > 7 years, clinical characteristics in CBC had a prominent impact on the prognosis of these patients, suggesting that interval time might determine the weight of clinical features on PBC and CBC in prognosis evaluation. While for patients with interval time within 3–7 years, the distribution of clinical features was between the above two-time dimensions (**Figure 3**).

### Bilateral characteristic DR model of BC based on interval time

**Figure 4:**
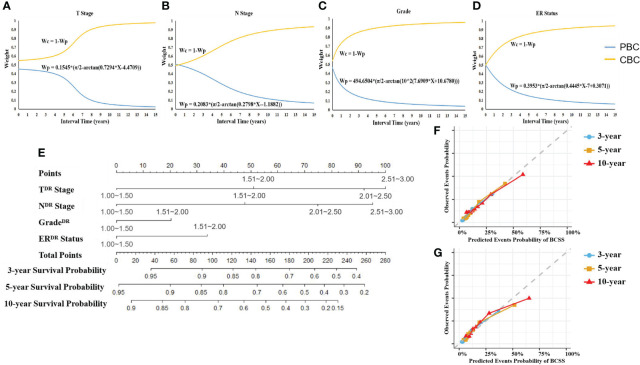
Weight of CIF curves on interval time and DR nomogram. **(A–D)** The weight of T stage **(A)**, N stage **(B)**, grade **(C)**, and ER status **(D)** belonging to PBC or CBC for prognostic prediction changed with interval time. CIF curves were created to identify the weight of PBC and CBC of the patient with specific interval time when using each character to predict BCCM. Wp, weight of PBC’s character. Wc, weight of CBC’s character. **(E)** DR prognostic nomogram for patients with MBBC. Competing risk dimensionality reduction nomogram for predicting the 3-, 5-, and 10-year probabilities of breast cancer–specific survival (BCSS). **(F, G)** Calibration plots for 3-, 5-, and 10-year probabilities of DR nomogram in the training **(F)** and validation **(G)** cohort. The solid line represents equality between the predicted and observed probabilities. With the dots close to the solid line, the plots reveal excellent agreement between the nomogram-predicted probabilities and actual observations.

Considering the important role of interval time in MBBC and the interference of interphase with other clinical factors on prognosis assessment, we illustrate the DR model dependent on interval time. Taking the T stage as an example (**Figure 4A**), we introduce a comprehensive indicator/index to describe the correlation between the bilateral staging:


$$T^{CI}=w_{c}\left(t\right)T^{c}+w_{p}\left(t\right)T^{p}$$

Where *w_c_* (*t*),*wp* (*t*) represent the incidence interval *t* dependent weighting function, which can be estimated by data fitting. We draw the CIF curve *f_ij_* of T stage with respect to interval, by univariate competing risk analysis (PBC defined as *i*, CBC defined as *j*), then calculate the *L* ^1^ norm of *f_ij_* for each subgroup


$$S_{ij}=\int_{0}^{R}f_{ij}\left(\text{τ}\right)d\text{τ}$$

For each interval *t*, we define the *L* ^1^ norm difference among all the subgroups as *D_p_*,


$$D_{p}=\sum_{j}\max\left|S_{i_{1}j}-S_{i_{2}j}\right|$$

$$i_{1}\neq i_{2}$$

Normalizing all the *D_p_*, and define it as the (PBC) weight *w_p_* for each interval *t*. By nonlinear fitting, we obtain the relationship between *w_p_* and *t*,


$$w_{p}=a\left(\frac{\text{π}}{2}-\arctan(bt+c\right)),$$

where a, b, and c are the coefficients. Then the weight for (CBC) is defined as


$$w_{c}=1-w_{p}$$

On this basis, we employed the same procedure to deal with N stage, tumor cell grade, and ER status (**Figures 4B** **–D**).

### DR nomogram

According to the DR CIF curve, we reduced the key clinical feature data of PBC and CBC in two-time dimensions to the unified dimension and assigned different weights. The weight of T stage on PBC and CBC, for example, a patient with interval of 5 years (T3 stage on PBC and T1 stage on CBC), was 0.37731 and 0.62269, respectively. Ulteriorly, referring to assignment score in **Table 2**, the DR of T stage (T^DR^ stage) = 3 × 0.37731 + 1 × 0.62269 = 1.75462, so T^DR^ stage was II stage (score: 1.51–2.0). The DR stage parameters were included in the multivariate analysis of competing risk, T^DR^ (*p*< 0.001), N^DR^ (*p*< 0.001), grade^DR^ (*p* = 0.028), ER^DR^ (*p*< 0.001), to generate a DR nomogram (**Figures 4E**; **Figure S1**).

The C-index of the nomogram was 0.773 (95% CI, 0.769–0.776) in training cohort (*n* = 8,869), and 0.819 (95% CI, 0.813–0.826) in validation cohort (*n* = 4435), respectively. In contrast to modeling for a single-spatial and temporal dimension in previous studies, DR nomogram was proved to have higher predictive power. Calibration plots revealed superb agreement between the nomogram-predicted probabilities and actual observations (**Figures 4F, G** **)**.

## Discussion

With regard to MBBC, no matter CBC is primary or metastatic, spatial–temporal heterogeneity between the PBC and CBC poses a significant challenge for assessing the prognosis and designing effective treatment regimens (26, 27). However, up to now, no studies have described the heterogeneous distribution and clonal evolution characteristics of these patients with MBBC (28, 29).

In this study, we collected samples and clinical data of BBC at disparate intervals from combined with large sample data from the SEER database, to analyze the clinical heterogeneous features and clonal evolution characteristics of MBBC from time and space dimensions. We verified that significant heterogeneity in genome (**Figure 2A**) and clinical features (**Table S2**) of BBC was widespread, especially for the diversity of driver gene mutation that was almost completely distinct between PBC and CBC. This significant heterogeneity poses a great challenge to the establishment of clinical prognostic models, which just based on unilateral lesion.

More importantly, we found that all of CBCs exhibited more different driver mutations and/or recurrent copy number aberrations than that in PBC, and the mutational diversity of CBC was significantly higher in patients with shorter interval time. In addition, a shorter interval time was significantly associated with a higher MATH ratio and poorer survival, mostly owe to the age of CBC diagnosis (**Figure 1E**), chemotherapy drugs (**Figure 2C**), and hereditary susceptibility (BRCA1/2 mutations) (25). It all suggested that, much shorter, an interval often indicated more malignant clonal evolution and interval time might have a vital influence on outcomes of MBBC.

Just since the time dimension and the weight of clinical features of bilateral lesions was crucial in the prognosis assessment of MBBC (**Figures 3A** **–D**), several previous clinical studies that tried to establish prognostic models based on clinical characteristics, just from one lesion (2, 3), cannot reflect the real prognosis. Some studies focused on the influence of worse characteristics on disease outcome (30). However, our study found that interval time plays an important role in prognosis of MBBC apart from clinical and molecular features. Thus, we included the interval time into account in our prognosis models (14).

To resolve the issue, we built a bilateral evaluation model that synchronously take heterogeneity of clinical features on both sides of the lesion into account for the first time, including T stage, N stage, grade, ER status, and interval time. Even so, the time dimension (interval time) was proven to have complex correlations with the other prognostic factors (*p*< 0.0001, **Figure 1E**, **Figure S2**), which could interfere with the predictive efficacy of the prognostic model. Thus, we reduced the time dimension dependent on the weight of clinical features of bilateral lesions at distant time node using CIF curves by crossing over with mathematics, to establish DR nomogram for actual observation for 3-, 5-, and 10-year BCCM, which was significantly better optimization of prognosis stratification than a traditional nomogram, and C-index improved by 0.05 and 0.06 in training cohort and internal validation cohort, respectively. In addition, this nomogram was only based on four basic clinical features, which greatly improves the clinical applicability of this model and facilitates clinical popularization. The application of the dimension reduction method could also extrapolate the prediction model to the clinical prediction of synchronous BC, and only the weight balance of occurrence of BBC is 0.5. A study-based SEER showed that the CBC were more and more likely to be detected at an early stage within short interval time (<= 1 years) and treated with mastectomy (4). The explanations of choosing mastectomy over breast-conserving surgery were complex and unclear, and we think with the help of the nomogram, more sensible therapeutic schedule will be made.

Validation of the nomogram is essential to avoid over-fitting and determine generalizability of prognostic model (31). In the current study, calibration plots showed optimal agreement between prediction and actual observation, guaranteeing the reliability and feasibility of the established nomogram (**Figures 4F** **, G**). The much higher C-index of the DR nomogram was revealed in internal validation cohort than that in the training cohort, indicating the effective repeatability. In the tentative external validation cohort from China (*n* = 89), the C-index was similar with the training cohort, suggested that the model was adaptable to the Asian population in spite of the small sample size (**Figure S3**). Even so, the validation of large sample and multicenter clinical data is still needed in the future.

On the other hand, whole-exome sequencing showed that gene mutations seemed to be completely different in PBC and CBC, hard to pin down the correlations with specific genetic mutations. However, the mutation signatures were all concentrated in the characteristics related to chemotherapeutic drugs alkylating agents, suggesting that the significance of drug stress selection in clone evolution (31, 32). This study provided an excellent *in vivo* model for improving the understanding of tumor evolution, which would guide clinical decision making to a certain extent. For example, the significance of chemotherapy elimination regimen for the malignant evolution of contralateral tumors and long-term outcome in patients with early BC should be considered.

However, our study still has the following limitations. First, given the low incidence of BBC with 0.22−3.08% in China (33), only 89 patients with BBC were included in this study among 25,119 cases from the First Hospital of Jilin University. Despite this external validation of the DR nomogram showed similar C-index with the training cohort, the small sample size still has the possibility of analysis bias. Even so, the validation of large sample and multicenter clinical data is still needed in the future to enhance the credibility of the results and applicability in clinical practice. Second, limited by the types of clinical and molecular factors included from SEER database, molecular indicators such as *BRCA* mutations were not included. Thus, it warrants an extend sample size with complete molecular, pathological, and clinical features to verify its clinical benefit.

In conclusion, we established and validated a novel DR nomogram for predicting BCCM of patients with MBBC. The clinicians could more precisely estimate the survival of individual patients and identify subgroups of patients who are in need of a specific treatment strategy by this nomogram.

## Data availability statement

The raw data supporting the conclusions of this article will be made available by the authors, without undue reservation.

## Ethics statement

The studies involving human participants were reviewed and approved by the Ethics Committee of the First Hospital of Jilin University. The patients/participants provided their written informed consent to participate in this study.

## Author contributions

Conception and design: LL and JC; Financial support: LL, JJ and JC. Administrative support: LL, JC, and HH; Provision of study materials or patients: JL, HG, HW, and ZL; Collection and assembly of data: JL, XY, HS, HW, and ZL; Data analysis and interpretation: LL, JL, JJ, ML, QY, and GL; Manuscript writing: LL, JL, JJ, ML, QY, and JC. Final approval of manuscript: All authors.

## Funding

This work was supported by National Natural Science Foundation of China grant (81874052) to JC; National Natural Science Foundation of China grant (82172690) and Translation-clinical Joint Foundation of the First Hospital of Jilin university (2020-ZL-06) to LL; Natural Science Foundation of Jilin Province (20210101481JC), and Shanghai Municipal Science and Technology Major Project (2021SHZDZX0103) to JJ.

## Acknowledgments

The authors thank Gang Li, Hongping Song and Yang Wang for their excellent assistance; they received no compensation for the contributions.

## Conflict of interest

Author QY was employed by Geneplus-Beijing.

The remaining authors declare that the research was conducted in the absence of any commercial or financial relationships that could be construed as a potential conflict of interest.

## Publisher’s note

All claims expressed in this article are solely those of the authors and do not necessarily represent those of their affiliated organizations, or those of the publisher, the editors and the reviewers. Any product that may be evaluated in this article, or claim that may be made by its manufacturer, is not guaranteed or endorsed by the publisher.
