# Supplementary figures and images for: Clonal evolution characteristics and reduced dimension prognostic model for non-metastatic metachronous bilateral breast cancer
Source: Front Oncol. 2022 Sep 29;12:963884. doi: 10.3389/fonc.2022.963884 (PMC9559188; doi:10.3389/fonc.2022.963884)

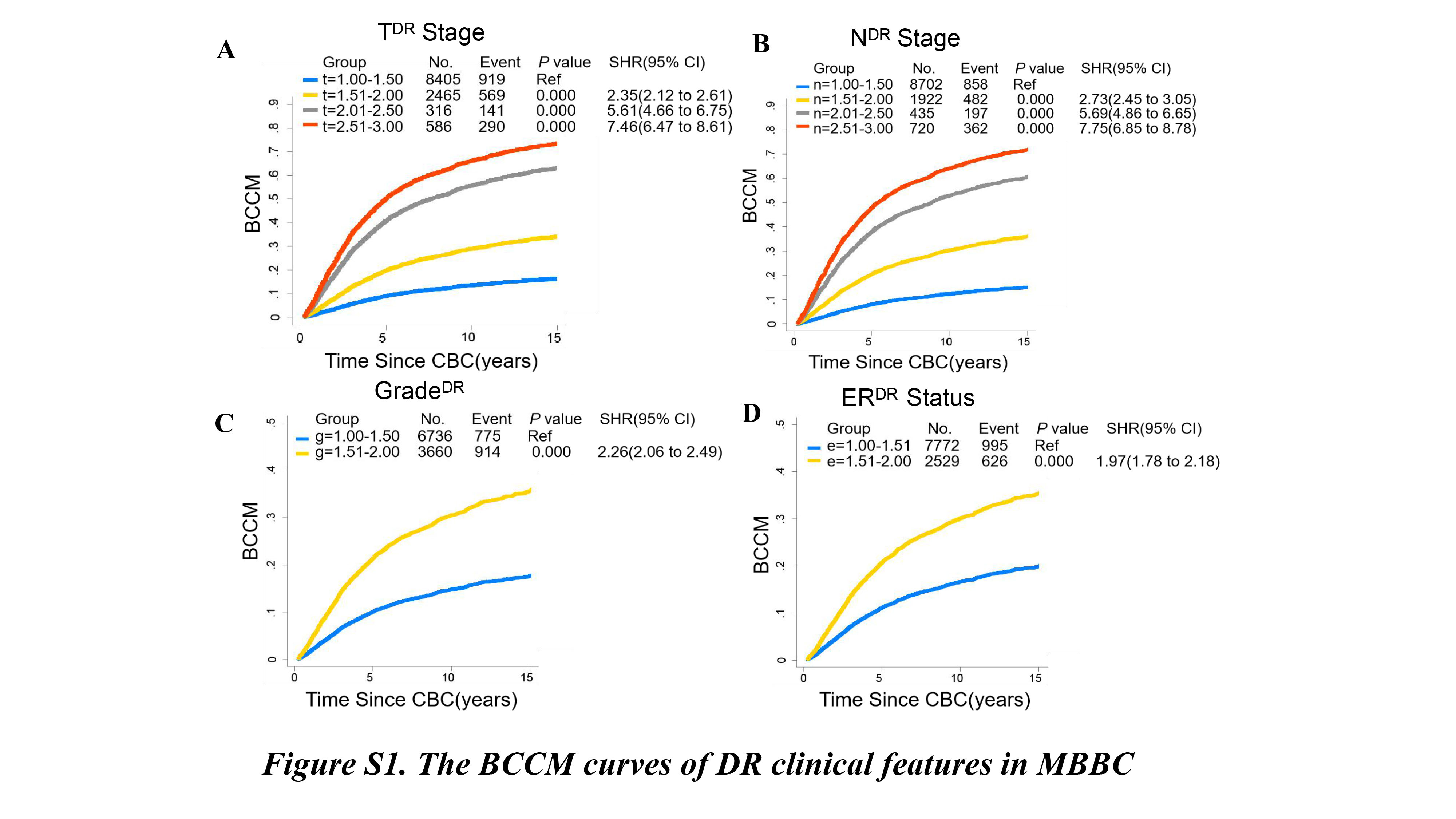

Supplement: Supplementary file 1 [file Image_1.jpeg]

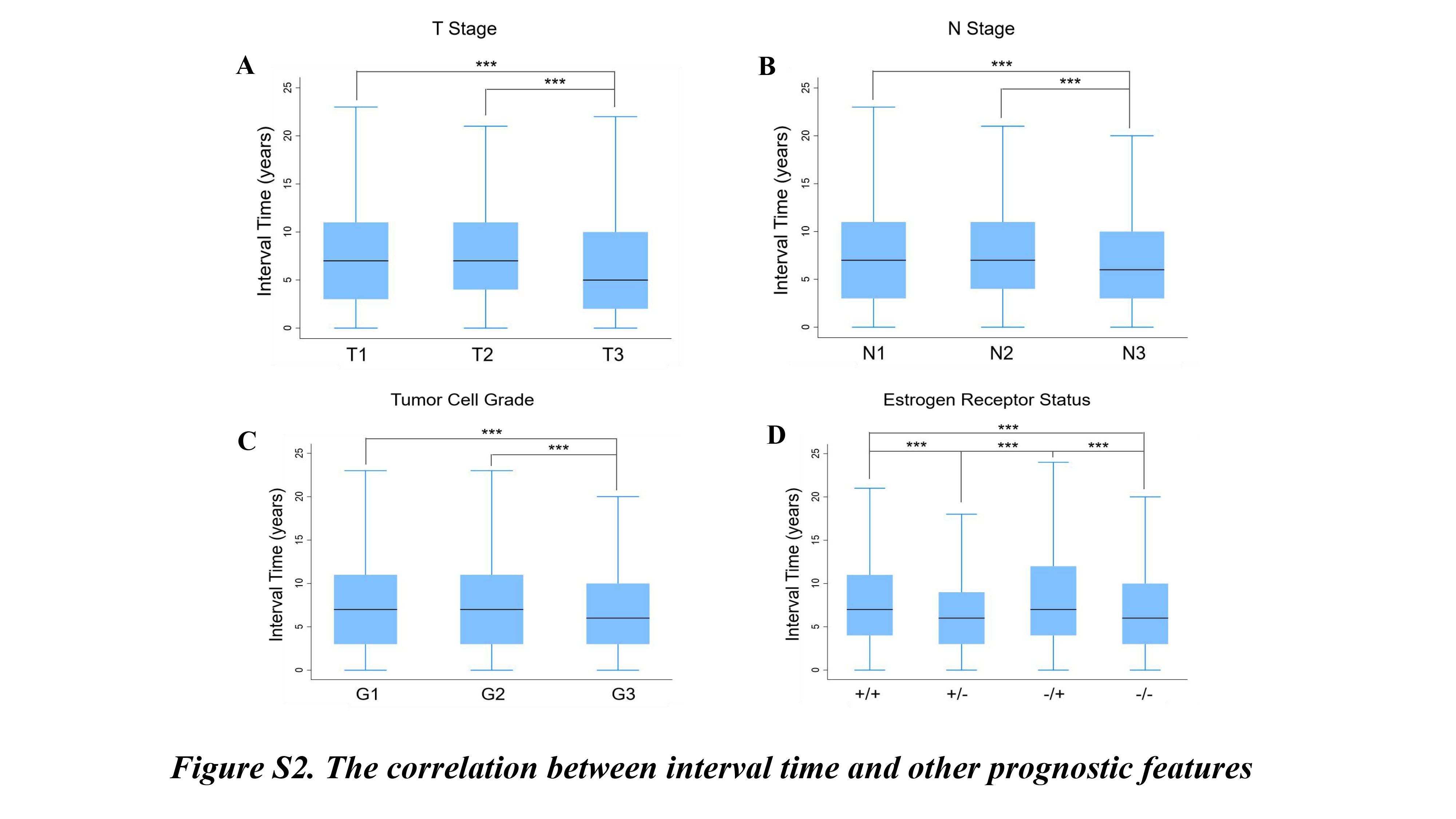

Supplement: Supplementary file 2 [file Image_2.jpeg]

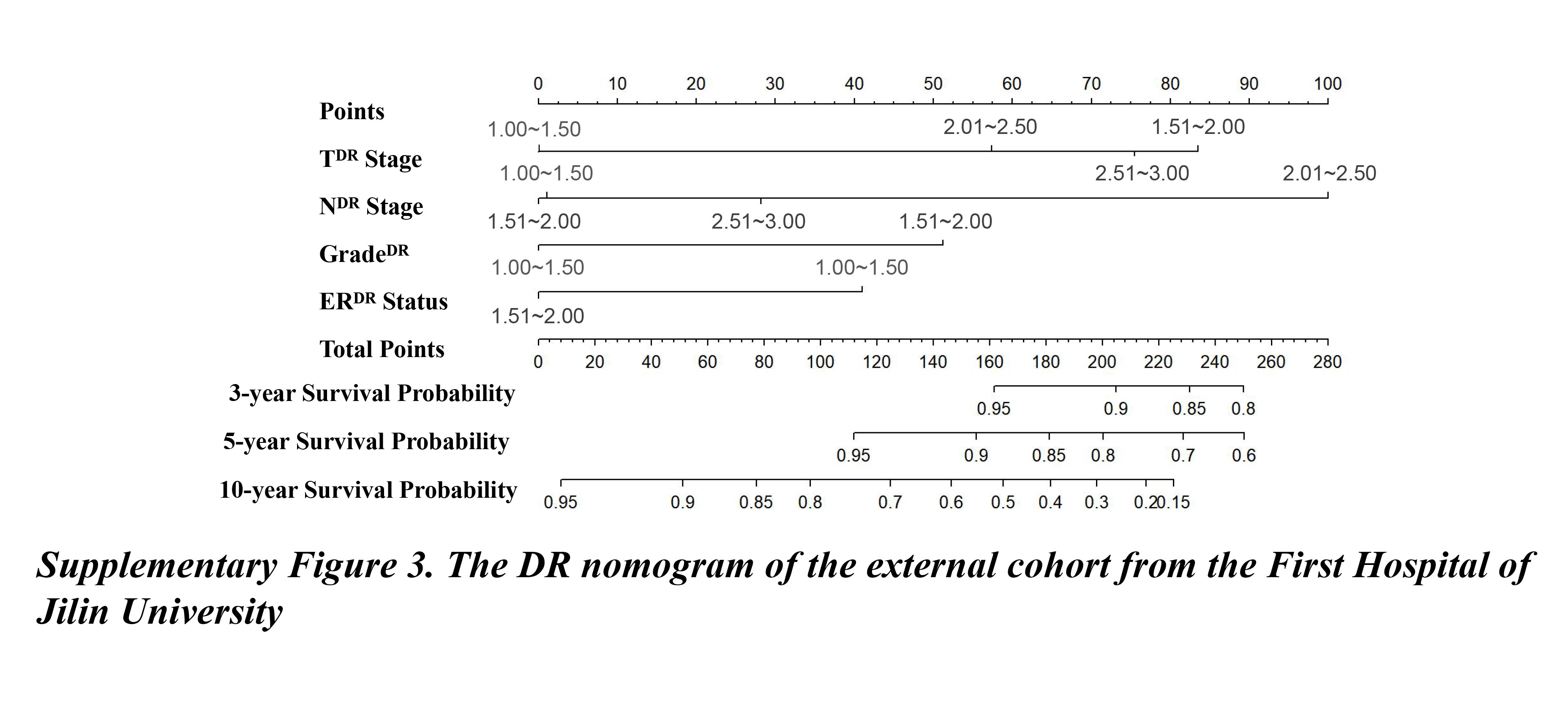

Supplement: Supplementary file 3 [file Image_3.jpeg]
