# Supplementary material for: Clonal evolution characteristics and reduced dimension prognostic model for non-metastatic metachronous bilateral breast cancer
Source: Front Oncol. 2022 Sep 29;12:963884. doi: 10.3389/fonc.2022.963884 (PMC9559188; doi:10.3389/fonc.2022.963884)
Supplement: Supplementary file 4 [file Table_1.docx]

***Supplementary Material***

**Supplementary Tables**

**Table S1. Clinical characteristics of breast cancer in SEER**（**1990-2015**）**(n=473909)**

| Character | No. of Patient（%） | | Character | No. of Patient（%） | |
| --- | --- | --- | --- | --- | --- |
| **Age, years** |  |  | **M Stage** |  |  |
| <= 40 | 36764 | (7.76) | Unknown | 16914 | (3.57) |
| 41-50 | 94970 | (20.04) | **Tumor Grade** |  |  |
| 51-60 | 112094 | (23.65) | I-II | 255719 | (53.96) |
| > 60 | 230031 | (48.54) | III-IV | 156350 | (32.99) |
| Unknown | 50 | (0.01) | Unknown | 61840 | (13.05) |
| **Sex** |  |  | **Pathological Type** |  |  |
| Female | 470785 | (99.34) | IDC | 336484 | (71.00) |
| Male | 3124 | (0.66) | ILC | 37228 | (7.86) |
| Race |  |  | Other | 100197 | (21.14) |
| White, non-Hispanic | 375213 | (79.17) | **Surgery Method** |  |  |
| Black, non-Hispanic | 46436 | (9.80) | BCS | 196149 | (41.39) |
| Other, mixed | 50129 | (10.58) | SM | 51017 | (10.77) |
| Unknown | 2131 | (0.45) | RM | 80540 | (16.99) |
| **Marital Status** |  |  | Other | 31444 | (6.64) |
| With Partner | 195835 | (41.32) | Unknown | 114759 | (24.22) |
| Without Partner | 257799 | (54.40) | **ER Status** |  |  |
| Unknown | 20275 | (4.28) | Positive | 327641 | (69.14) |
| **T Stage** |  |  | Negative | 86557 | (18.26) |
| T1 | 252177 | (53.21) | Borderline | 1540 | (0.32) |
| T2 | 123169 | (25.99) | Unknown | 58171 | (12.27) |
| T3-T4 | 35651 | (7.52) | **PR Status** |  |  |
| Unknown | 62912 | (13.28) | Positive | 278707 | (58.81) |
| **N Stage** |  |  | Negative | 128293 | (27.07) |
| N0 | 278484 | (58.76) | Borderline | 2569 | (0.54) |
| N1 | 98262 | (20.73) | Unknown | 64340 | (13.58) |
| N2-N3 | 50626 | (10.68) | **HER2 Status** |  |  |
| Unknown | 46537 | (9.82) | Positive | 20014 | (4.22) |
| **M Stage** |  |  | Negative | 104653 | (22.08) |
| M0 | 433184 | (91.41) | Borderline | 2845 | (0.60) |
| M1 | 23811 | (5.02) | Unknown | 346397 | (73.09) |

Abbreviations: With Partner, with partner at diagnosis (married, unmarried or domestic partner, same sex or opposite sex partner); Without-Partner, without partner at diagnosis (single, divorced, widowed, separated); T, tumor; N: node; IDC, invasive ductal carcinoma; ILC, invasive lobular carcinoma; BCS, breast-conserving surgery; SM, simple mastectomy; RM, radical mastectomy; ER, estrogen receptor; PR, progesterone receptor; HER2, human epidermal growth factor receptor.

**Table S2. Clinical characteristics of MBBC in the SEER database**

|  |  |  |  |  | Marginal Homogeneity Test | | | Wilcoxon Test | |
| --- | --- | --- | --- | --- | --- | --- | --- | --- | --- |
|  |  |  |  |  | Std. MH Statistic | *P* Value | | Z | *P* Value |
| **Marital Status** | CBC | | |  |  |  | |  |  |
| PBC | With Partner | Without Partner |  | Total |  |  | |  |  |
| With Partner | 4371 | 382 |  | 4753 | -22.454 | < 0.001 | | -22.454 | < 0.001 |
| Without Partner | 1304 | 6359 |  | 7663 |  |  | |  |  |
| Total | 5675 | 6741 |  | 12416 |  |  | |  |  |
| **T Stage** | CBC | | |  |  |  | |  |  |
| PBC | T1 | T2 | T3-T4 | Total |  |  | |  |  |
| T1 | 6069 | 1438 | 285 | 7792 | 11.953 | < 0.001 | | -11.828 | < 0.001 |
| T2 | 2169 | 757 | 237 | 3163 |  |  | |  |  |
| T3-T4 | 437 | 197 | 183 | 817 |  |  | |  |  |
| Total | 8675 | 2392 | 705 | 11772 |  |  | |  |  |
| **N Stage** | CBC | | | |  |  | |  |  |
| PBC | N0 | N1 | N2-N3 | Total |  |  | |  |  |
| N0 | 6740 | 1236 | 499 | 8475 | 6.594 | < 0.001 | | -6.396 | < 0.001 |
| N1 | 1620 | 404 | 230 | 2254 |  |  | |  |  |
| N2-N3 | 615 | 194 | 241 | 1050 |  |  | |  |  |
| Total | 8975 | 1834 | 970 | 11779 |  |  | |  |  |
| **Tumor Grade** | CBC | | |  |  | |  |  |  |
| PBC | I-II | III-IV |  | Total |  | |  |  |  |
| I-II | 4738 | 1562 |  | 6300 | -7.307 | | < 0.001 | -7.307 | < 0.001 |
| III-IV | 1998 | 2098 |  | 4096 |  | |  |  |  |
| Total | 6736 | 3660 |  | 10396 |  | |  |  |  |
| (continued on following page) | | | | | | | | | |

**Table S2. Clinical characteristics of MBBC in the SEER database (continued)**

|  |  |  |  |  | Marginal Homogeneity Test | | Wilcoxon Test | |
| --- | --- | --- | --- | --- | --- | --- | --- | --- |
|  |  |  |  |  | Std. MH Statistic | *P* Value | Z | *P* Value |
| **Pathological Type** | CBC | | |  |  |  |  |  |
| PBC | IDC | ILC | Other | Total |  |  |  |  |
| IDC | 6841 | 825 | 1686 | 9352 | 2.300 | 0.021 | -3.022 | 0.003 |
| ILC | 536 | 278 | 177 | 991 |  |  |  |  |
| Other | 1909 | 313 | 739 | 2961 |  |  |  |  |
| Total | 9286 | 1416 | 2602 | 13304 |  |  |  |  |
| **Surgery Method** | CBC | | | |  |  |  |  |
| PBC | BCS | SM | RM | Total |  |  |  |  |
| BCS | 2699 | 814 | 541 | 4054 | -8.977 | < 0.001 | -8.895 | < 0.001 |
| SM | 124 | 332 | 154 | 610 |  |  |  |  |
| RM | 330 | 619 | 776 | 1725 |  |  |  |  |
| Total | 3153 | 1765 | 1471 | 6389 |  |  |  |  |
| **ER Status** | CBC | | |  |  |  |  |  |
| PBC | Positive | Negative |  | Total |  |  |  |  |
| Positive | 6286 | 1279 |  | 7565 | -3.827 | < 0.001 | -3.827 | < 0.001 |
| Negative | 1480 | 1256 |  | 2736 |  |  |  |  |
| Total | 7766 | 2535 |  | 10301 |  |  |  |  |
| **PR Status** | CBC | | |  |  |  |  |  |
| PBC | Positive | Negative |  | Total |  |  |  |  |
| Positive | 4290 | 2213 |  | 6503 | 10.067 | < 0.001 | -10.067 | < 0.001 |
| Negative | 1592 | 1769 |  | 3361 |  |  |  |  |
| Total | 5882 | 3982 |  | 9864 |  |  |  |  |
| **HER2 Status** | CBC | | |  |  |  |  |  |
| PBC | Positive | Negative |  | Total |  |  |  |  |
| Positive | 23 | 26 |  | 49 | -3.130 | 0.002 | -3.130 | 0.002 |
| Negative | 54 | 373 |  | 427 |  |  |  |  |
| Total | 77 | 399 |  | 476 |  |  |  |  |

Abbreviations: PBC, primary breast cancer; CBC, contralateral breast cancer; Std. MH Statistic, standard Marginal Homogeneity statistic. With Partner, with partner at diagnosis (married, unmarried or domestic partner, same sex or opposite sex partner); Without-Partner, without partner at diagnosis (single, divorced, widowed, separated); T, tumor; N: node; IDC, invasive ductal carcinoma; ILC, invasive lobular carcinoma; BCS, breast-conserving surgery; SM, simple mastectomy; RM, radical mastectomy; ER, estrogen receptor; PR, progesterone receptor; HER2, human epidermal growth factor receptor 2.

**Table S3.** **Baseline characteristics of 2:1 MBBC cohort**

|  | All Patients  N=13304  No. (%) | Training Cohort  N=8869  No. (%) | Validation Cohort  N=4435  No. (%) | *P* Value |
| --- | --- | --- | --- | --- |
| **Age at Diagnosis of CBC (years)** |  |  |  | 0.144 |
| <= 40 | 411 (3.09%) | 284 (3.20%) | 127 (2.86%) |  |
| 41-50 | 1493 (11.22%) | 1020 (11.50%) | 473 (10.67%) |  |
| 51-60 | 2739 (20.59%) | 1848 (20.84%) | 891 (20.09%) |  |
| > 60 | 8661 (65.10%) | 5717 (64.46%) | 2944 (66.38%) |  |
| **Interval Time（years）** |  |  |  |  |
| <= 7 | 7551 (56.76%) | 5053 (56.97%) | 2498 (56.32%) | 0.481 |
| > 7 | 5753 (43.24%) | 3816 (43.03%) | 1937 (43.68%) |  |
| <= 3 | 3131 (23.53%) | 2110 (23.79%) | 1021 (23.02%) | 0.329 |
| > 3 | 10173 (76.47%) | 6759 (76.21%) | 3414 (76.98%) |  |
| **Race** |  |  |  | 0.977 |
| White,  non-Hispanic | 10731 (80.66%) | 7150 (80.62%) | 3581 (80.74%) |  |
| Black, non-Hispanic | 1308 (9.83%) | 871 (9.82%) | 437 (9.85%) |  |
| Other, mixed | 1261 (9.48%) | 845 (9.53%) | 416 (9.38%) |  |
| Unknown | 4 (0.03%) | 3 (0.03%) | 1 (0.02%) |  |
| **Marital Status** |  |  |  | 0.079 |
| Non-P/Non-P | 4371 (32.85%) | 2924 (32.97%) | 1447 (32.63%) |  |
| Non-P/With-P | 382 (2.87%) | 263 (2.97%) | 119 (2.68%) |  |
| With-P/Non-P | 1304 (9.80%) | 832 (9.38%) | 472 (10.64%) |  |
| With-P/With-P | 6359 (47.80%) | 4281 (48.27%) | 2078 (46.85%) |  |
| Unknown | 888 (6.67%) | 569 (6.42%) | 319 (7.19%) |  |
| **T Stage** |  |  |  | 0.791 |
| T1/T1 | 6069 (45.62%) | 4050 (45.66%) | 2019 (45.52%) |  |
| T1/T2 | 1438 (10.81%) | 965 (10.88%) | 473 (10.67%) |  |
| T1/T3-T4 | 285 (2.14%) | 186 (2.10%) | 99 (2.23%) |  |
| T2/T1 | 2169 (16.30%) | 1446 (16.30%) | 723 (16.30%) |  |
| T2/T2 | 757 (5.69%) | 502 (5.66%) | 255 (5.75%) |  |
| T2/T3-T4 | 237 (1.78%) | 161 (1.82%) | 76 (1.71%) |  |
| T3-T4/TI | 437 (3.28%) | 301 (3.39%) | 136 (3.07%) |  |
| T3-T4/T2 | 197 (1.48%) | 140 (1.58%) | 57 (1.29%) |  |
| T3-T4/T3-T4 | 183 (1.38%) | 115 (1.30%) | 68 (1.53%) |  |
| (continued on following page) | | | | |

**Table S3.** **Baseline characteristics of 2:1 MBBC cohort (continued)**

|  | All Patients  N=13304  No. (%) | Training Cohort  N=8869  No. (%) | Validation Cohort  N=4435  No. (%) | *P* Value |
| --- | --- | --- | --- | --- |
| Unknown | 1532 (11.52%) | 1003 (11.31%) | 529 (11.93%) |  |
| **N Stage** |  |  |  | 0.911 |
| N0/N0 | 6740 (50.66%) | 4516 (50.92%) | 2224 (50.15%) |  |
| N0/N1 | 1236 (9.29%) | 826 (9.31%) | 410 (9.24%) |  |
| N0/N2-N3 | 499 (3.75%) | 341 (3.84%) | 158 (3.56%) |  |
| N1/N0 | 1620 (12.18%) | 1072 (12.09%) | 548 (12.36%) |  |
| N1/N1 | 404 (3.04%) | 266 (3.00%) | 138 (3.11%) |  |
| N1/N2-N3 | 230 (1.73%) | 152 (1.71%) | 78 (1.76%) |  |
| N2-N3/N0 | 615 (4.62%) | 409 (4.61%) | 206 (4.64%) |  |
| N2-N3/N1 | 194 (1.46%) | 121 (1.36%) | 73 (1.65%) |  |
| N2-N3/N2-N3 | 241 (1.81%) | 165 (1.86%) | 76 (1.71%) |  |
| Unknown | 1525 (11.46%) | 1001 (11.29%) | 524 (11.82%) |  |
| **Tumor Grade** |  |  |  | 0.441 |
| I-II/I-II | 4738 (35.61%) | 3136 (35.36%) | 1602 (36.12%) |  |
| I-II/III-IV | 1562 (11.74%) | 1018 (11.48%) | 544 (12.27%) |  |
| III-IV/I-II | 1998 (15.02%) | 1338 (15.09%) | 660 (14.88%) |  |
| III-IV/III-IV | 2098 (15.77%) | 1408 (15.88%) | 690 (15.56%) |  |
| Unknown | 2908 (21.86%) | 1969 (22.20%) | 939 (21.17%) |  |
| **Pathological Type** |  |  |  | 0.344 |
| IDC/IDC | 6841 (51.42%) | 4560 (51.42%) | 2281 (51.43%) |  |
| IDC/ILC | 825 (6.20%) | 541 (6.10%) | 284 (6.40%) |  |
| IDC/Other | 1686 (12.67%) | 1135 (12.80%) | 551 (12.42%) |  |
| ILC/IDC | 536 (4.03%) | 340 (3.83%) | 196 (4.42%) |  |
| ILC/ILC | 278 (2.09%) | 179 (2.02%) | 99 (2.23%) |  |
| ILC/Other | 177 (1.33%) | 118 (1.33%) | 59 (1.33%) |  |
| Other/IDC | 1909 (14.35%) | 1280 (14.43%) | 629 (14.18%) |  |
| Other/ILC | 313 (2.35%) | 227 (2.56%) | 86 (1.94%) |  |
| Other/Other | 739 (5.55%) | 489 (5.51%) | 250 (5.64%) |  |
| **Surgery Method** |  |  |  | 0.005 |
| BCM/BCM | 2699 (20.29%) | 1743 (19.65%) | 956 (21.56%) |  |
| BCM/SM | 814 (6.12%) | 519 (5.85%) | 295 (6.65%) |  |
| BCM/RM | 541 (4.07%) | 365 (4.12%) | 176 (3.97%) |  |
| SM/BCM | 124 (0.93%) | 76 (0.86%) | 48 (1.08%) |  |
| SM/SM | 332 (2.50%) | 245 (2.76) | 87 (1.96%) |  |
| (continued on following page) | | | | |

**Table S3.** **Baseline characteristics of 2:1 MBBC cohort (continued)**

|  | All Patients  N=13304  No. (%) | Training Cohort  N=8869  No. (%) | Validation Cohort  N=4435  No. (%) | *P* Value |
| --- | --- | --- | --- | --- |
| SM/RM | 154 (1.16%) | 112 (1.26%) | 42 (0.95%) |  |
| RM/BCM | 330 (2.48%) | 223 (2.51%) | 107 (2.41%) |  |
| RM/SM | 619 (4.65%) | 431 (4.86%) | 188 (4.24%) |  |
| RM/RM | 776 (5.83%) | 529 (5.96%) | 247 (5.57%) |  |
| Unknown | 6915 (51.98%) | 4626 (52.16%) | 2289 (51.61%) |  |
| **ER Status** |  |  |  | 0.496 |
| +/+ | 6286 (47.25%) | 4173 (47.05%) | 2113 (47.64%) |  |
| +/- | 1279 (9.61%) | 838 (9.45%) | 441 (9.94%) |  |
| -/+ | 1480 (11.12%) | 983 (11.08%) | 497 (11.21%) |  |
| -/- | 1256 (9.44%) | 862 (9.72%) | 394 (8.88%) |  |
| Unknown/  Borderline | 3003 (22.57%) | 2013 (22.70%) | 990 (22.32%) |  |
| **PR Status** |  |  |  | 0.488 |
| +/+ | 4290 (32.25%) | 2862 (32.27%) | 1428 (32.20%) |  |
| +/- | 2213 (16.63%) | 1447 (16.32%) | 766 (17.27%) |  |
| -/+ | 1592 (11.97%) | 1056 (11.91%) | 536 (12.09%) |  |
| -/- | 1769 (13.30%) | 1205 (13.59%) | 564 (12.72%) |  |
| Unknown/  Borderline | 3440 (25.86%) | 2299 (25.92%) | 1141 (25.73%) |  |
| **HER2 Status** |  |  |  | 0.626 |
| +/+ | 23 (0.17%) | 14 (0.16%) | 9 (0.20%) |  |
| +/- | 26 (0.20%) | 20 (0.23%) | 6 (0.14%) |  |
| -/+ | 54 (0.41%) | 33 (0.37%) | 21 (0.47%) |  |
| -/- | 373 (2.80%) | 244 (2.75%) | 129 (2.91%) |  |
| Unknown/  Borderline | 12828 (96.42%) | 8558 (96.49%) | 4270 (96.28%) |  |

Abbreviations: MBBC, metachronous bilateral breast cancer; N, number of patients in total cohort, training cohort and validation cohort; Non-P, without partner at diagnosis (single, divorced, widowed, separated); With-P, with partner at diagnosis (married, unmarried or domestic partner, same sex or opposite sex partner); T, tumor; N: node; IDC, invasive ductal carcinoma; ILC, invasive lobular carcinoma; BCS, breast-conserving surgery; SM, simple mastectomy; RM, radical mastectomy; ER, estrogen receptor; PR, progesterone receptor; HER2, human epidermal growth factor receptor 2; +, positive; -, negative.

**Table S4.** **Competing risk model for training cohort**

| Variable | Univariate Analysis | | | | Multivariate Analysis | | | |
| --- | --- | --- | --- | --- | --- | --- | --- | --- |
|  | *P* Value | Sub-  Distri-  bution HR | 95%CI low | 95%  CI upp | *P* Value | Sub-  Distri-  bution HR | 95%  CI low | 95%  CI upp |
| Age at Diagnosis of CBC (years) | | | | |  |  |  |  |
| <= 40 | **Ref** |  |  |  | **Ref** |  |  |  |
| 41-50 | < 0.001 | 0.626 | 0.500 | 0.784 | 0.807 | 0.930 | 0.521 | 1.661 |
| 51-60 | < 0.001 | 0.456 | 0.368 | 0.566 | 0.491 | 1.219 | 0.694 | 2.141 |
| >60 | < 0.001 | 0.348 | 0.284 | 0.425 | 0.652 | 1.138 | 0.649 | 1.994 |
| Interval Time (years) | | | | |  |  |  |  |
| Continuous Variable form | | |  |  | **<0.001** | **0.927** | **0.893** | **0.962** |
| <= 7 | **Ref** |  |  |  |  |  |  |  |
| > 7 | < 0.001 | 0.563 | 0.502 | 0.631 |  |  |  |  |
| <= 3 | **Ref** |  |  |  |  |  |  |  |
| > 3 | < 0.001 | 0.621 | 0.559 | 0.690 |  |  |  |  |
| Race |  |  |  |  |  |  |  |  |
| White,  non-Hispanic | **Ref** |  |  |  | **Ref** |  |  |  |
| Black, non-Hispanic | < 0.001 | 1.617 | 1.397 | 1.872 | 0.280 | 0.829 | 0.590 | 1.165 |
| Other, mixed | 0.771 | 0.974 | 0.814 | 1.164 | 0.130 | 1.330 | 0.919 | 1.925 |
| Marital Status |  |  |  |  |  |  |  |  |
| Non-P/Non-P | **Ref** |  |  |  | **Ref** |  |  |  |
| Non-P/With-P | 0.300 | 0.847 | 0.619 | 1.160 | 0.464 | 0.783 | 0.408 | 1.505 |
| With-P/Non-P | 0.098 | 0.852 | 0.705 | 1.030 | 0.205 | 0.710 | 0.417 | 1.206 |
| With-P/With-P | 0.001 | 0.836 | 0.750 | 0.932 | 0.062 | 0.797 | 0.629 | 1.011 |
| (continued on following page) | | | | | | | | |

**Table S4.** **Competing risk model for training cohort (continued)**

| Variable | Univariate Analysis | | | | Multivariate Analysis | | | |
| --- | --- | --- | --- | --- | --- | --- | --- | --- |
|  | *P* Value | Sub-  Distri-  bution HR | 95%CI low | 95%  CI upp | *P* Value | Sub-  Distri-  bution HR | 95%  CI low | 95%  CI upp |
| T Stage | | | | |  |  |  |  |
| T1/T1 | **Ref** |  |  |  | **Ref** |  |  |  |
| T1/T2 | < 0.001 | 2.300 | 1.936 | 2.733 | **< 0.001** | **2.181** | **1.516** | **3.138** |
| T1/T3-T4 | < 0.001 | 6.677 | 5.189 | 8.591 | **< 0.001** | **5.634** | **3.269** | **9.710** |
| T2/T1 | < 0.001 | 1.926 | 1.645 | 2.255 | **0.001** | **1.860** | **1.307** | **2.647** |
| T2/T2 | < 0.001 | 3.332 | 2.734 | 4.061 | **< 0.001** | **3.112** | **2.083** | **4.650** |
| T2/T3-T4 | < 0.001 | 9.650 | 7.413 | 12.562 | **< 0.001** | **4.652** | **2.292** | **9.441** |
| T3-T4/TI | < 0.001 | 2.941 | 2.281 | 3.792 | **0.001** | **2.537** | **1.468** | **4.387** |
| T3-T4/T2 | < 0.001 | 6.213 | 4.633 | 8.332 | **< 0.001** | **3.194** | **1.748** | **5.836** |
| T3-T4/T3-T4 | < 0.001 | 14.192 | 10.705 | 18.815 | **0.002** | **3.414** | **1.590** | **7.332** |
| N Stage | | | | |  |  |  |  |
| N0/N0 | **Ref** |  |  |  | **Ref** |  |  |  |
| N0/N1 | < 0.001 | 2.170 | 1.805 | 2.608 | **0.020** | **1.553** | **1.071** | **2.251** |
| N0/N2-N3 | < 0.001 | 5.862 | 4.825 | 7.122 | **< 0.001** | **3.223** | **2.048** | **5.071** |
| N1/N0 | < 0.001 | 1.805 | 1.507 | 2.161 | **0.022** | **1.505** | **1.059** | **2.138** |
| N1/N1 | < 0.001 | 3.991 | 3.147 | 5.061 | **< 0.001** | **2.847** | **1.832** | **4.424** |
| N1/N2-N3 | < 0.001 | 9.578 | 7.649 | 11.994 | **< 0.001** | **3.400** | **1.995** | **5.795** |
| N2-N3/N0 | < 0.001 | 3.079 | 2.490 | 3.808 | **0.004** | **2.096** | **1.271** | **3.457** |
| N2-N3/N1 | < 0.001 | 5.347 | 3.906 | 7.319 | **< 0.001** | **3.506** | **1.784** | **6.889** |
| N2-N3/N2-N3 | < 0.001 | 14.244 | 11.411 | 17.781 | **< 0.001** | **5.567** | **3.117** | **9.945** |
| (continued on following page) | | | | | | | | |

**Table S4.** **Competing risk model for training cohort (continued)**

| Variable | Univariate Analysis | | | | Multivariate Analysis | | | |
| --- | --- | --- | --- | --- | --- | --- | --- | --- |
|  | *P* Value | Sub-  Distri-  bution HR | 95%CI low | 95%  CI upp | *P* Value | Sub-  Distri-  bution HR | 95%  CI low | 95%  CI upp |
| Tumor Grade | | | | |  |  |  |  |
| I-II/I-II | **Ref** |  |  |  | **Ref** |  |  |  |
| I-II/  III- IV | < 0.001 | 1.938 | 1.635 | 2.297 | 0.104 | 1.309 | 0.946 | 1.812 |
| III-IV/  I-II | 0.005 | 1.284 | 1.078 | 1.529 | 0.551 | 1.115 | 0.780 | 1.592 |
| III-IV/  III-IV | < 0.001 | 2.724 | 2.358 | 3.146 | **0.007** | **1.612** | **1.140** | **2.278** |
| Pathological Type | | | | |  |  |  |  |
| IDC/  IDC | **Ref** |  |  |  | **Ref** |  |  |  |
| IDC/  ILC | 0.558 | 1.063 | 0.866 | 1.306 | 0.801 | 0.931 | 0.535 | 1.622 |
| IDC/  Other | 0.837 | 1.017 | 0.870 | 1.188 | 0.856 | 0.965 | 0.658 | 1.416 |
| ILC/  IDC | 0.836 | 1.027 | 0.795 | 1.328 | 0.472 | 1.282 | 0.651 | 2.525 |
| ILC/  ILC | 0.004 | 1.563 | 1.158 | 2.109 | 0.811 | 0.900 | 0.379 | 2.138 |
| ILC/  Other | 0.028 | 1.458 | 1.043 | 2.039 | 0.314 | 1.461 | 0.698 | 3.059 |
| Other/  IDC | 0.823 | 1.017 | 0.878 | 1.178 | **0.007** | **1.542** | **1.128** | **2.108** |
| Other/  ILC | 0.509 | 1.113 | 0.810 | 1.529 | 0.107 | 1.586 | 0.905 | 2.780 |
| Other/  Other | 0.249 | 1.133 | 0.916 | 1.401 | 0.105 | 1.440 | 0.926 | 2.238 |
| Surgery Method | | | | |  |  |  |  |
| BCM/  BCM | **Ref** |  |  |  | **Ref** |  |  |  |
| BCM/  SM | 0.177 | 1.254 | 0.903 | 1.742 | 0.912 | 0.977 | 0.652 | 1.466 |
| BCM/  RM | < 0.001 | 2.424 | 1.838 | 3.197 | 0.604 | 1.110 | 0.748 | 1.649 |
| (continued on following page) | | | | | | | |  |

**Table S4.** **Competing risk model for training cohort (continued)**

| Variable | Univariate Analysis | | | | Multivariate Analysis | | | |
| --- | --- | --- | --- | --- | --- | --- | --- | --- |
|  | *P* Value | Sub-  Distri-  bution HR | 95%CI low | 95%  CI upp | *P* Value | Sub-  Distri-  bution HR | 95%  CI low | 95%  CI upp |
| SM/  BCM | 0.441 | 1.344 | 0.634 | 2.850 | 0.880 | 0.927 | 0.349 | 2.464 |
| SM/SM | 0.375 | 0.781 | 0.452 | 1.349 | 0.139 | 0.575 | 0.276 | 1.197 |
| SM/RM | < 0.001 | 2.625 | 1.708 | 4.035 | 0.802 | 1.080 | 0.592 | 1.972 |
| RM/  BCM | < 0.001 | 2.289 | 1.627 | 3.221 | 0.729 | 1.094 | 0.656 | 1.825 |
| RM/SM | 0.002 | 1.650 | 1.204 | 2.261 | 0.818 | 0.948 | 0.601 | 1.495 |
| RM/RM | < 0.001 | 3.061 | 2.437 | 3.845 | 0.634 | 1.086 | 0.772 | 1.529 |
| ER Status | | | | |  |  |  |  |
| +/+ | **Ref** |  |  |  | **Ref** |  |  |  |
| +/- | < 0.001 | 1.779 | 1.505 | 2.102 | 0.155 | 1.264 | 0.915 | 1.745 |
| -/+ | 0.458 | 0.928 | 0.762 | 1.131 | 0.136 | 0.748 | 0.510 | 1.096 |
| -/- | < 0.001 | 2.339 | 2.003 | 2.730 | 0.091 | 1.377 | 0.950 | 1.997 |
| PR Status | | | | |  |  |  |  |
| +/+ | **Ref** |  |  |  |  |  |  |  |
| +/- | < 0.001 | 1.680 | 1.440 | 1.960 |  |  |  |  |
| -/+ | 0.183 | 0.866 | 0.701 | 1.070 |  |  |  |  |
| -/- | < 0.001 | 2.103 | 1.799 | 2.460 |  |  |  |  |
| HER2 Status | | | | |  |  |  |  |
| +/+ | **Ref** |  |  |  |  |  |  |  |
| +/- | - | - | - | - |  |  |  |  |
| -/+ | 0.124 | 0.143 | 0.012 | 1.702 |  |  |  |  |
| -/- | 0.123 | 0.308 | 0.069 | 1.375 |  |  |  |  |

Abbreviations: Sub-Distribution HR, subdistribution hazard ratio; CI, confidence interval; low, lower bound of confidence interval; upp, upper bound of confidence interval; CBC, contralateral breast cancer; Ref, reference; Non-P, without partner at diagnosis (single, divorced, widowed, separated); With-P, with partner at diagnosis (married, unmarried or domestic partner, same sex or opposite sex partner); T, tumor; N: node; IDC, invasive ductal carcinoma; ILC, invasive lobular carcinoma; BCS, breast-conserving surgery; SM, simple mastectomy; RM, radical mastectomy; ER, estrogen receptor; PR, progesterone receptor; HER2, human epidermal growth factor receptor 2; +, positive; -, negative.

**Table S5. Multivariate analysis of competing risk model after dimension reduction**

| Variable | No. (%)  (N=8869) | Multivariate Analysis | | | |
| --- | --- | --- | --- | --- | --- |
|  |  | P Value | Sub-  Distri-  bution HR | 95%  CI low | 95%  CI upp |
| T^DR^ Stage |  |  |  |  |  |
| T^DR^1 | 5603 (62.47%) | Ref |  |  |  |
| T^DR^2 | 1661 (18.52%) | < 0.001 | 1.900 | 1.455 | 2.482 |
| T^DR^3 | 216 (2.41%) | < 0.001 | 3.058 | 1.919 | 4.871 |
| T^DR^4 | 486 (5.42%) | < 0.001 | 3.600 | 2.260 | 5.736 |
| Unknown | 1003 (11.18%) |  |  |  |  |
| N^DR^ stage |  |  |  |  |  |
| N^DR^1 | 5820 (65.62%) | Ref |  |  |  |
| N^DR^2 | 1278 (14.41%) | < 0.001 | 1.825 | 1.386 | 2.404 |
| N^DR^3 | 292 (3.29%) | < 0.001 | 2.666 | 1.750 | 4.062 |
| N^DR^4 | 478 (5.39%) | < 0.001 | 3.359 | 2.259 | 4.993 |
| Unknown | 1001 (11.29%) |  |  |  |  |
| Grade^DR^ |  |  |  |  |  |
| Grade^DR^1 | 4474 (50.45%) | Ref |  |  |  |
| Grade^DR^2 | 2426 (27.35%) | 0.028 | 1.321 | 1.031 | 1.694 |
| Unknown | 1969 (22.20%) |  |  |  |  |
| ER^DR^ Status |  |  |  |  |  |
| ER^DR^1 | 5160 (58.18%) | Ref |  |  |  |
| ER^DR^2 | 1696 (19.12%) | < 0.001 | 1.588 | 1.228 | 2.053 |
| Unknown | 2013 (22.70%) |  |  |  |  |

Abbreviations: No., number (N); Sub-Distribution HR, subdistribution hazard ratio; CI, confidence interval; low, lower bound of confidence interval; upp, upper bound of confidence interval; Ref, reference; DR: dimension reduction; T^DR^, revised T (tumor) stage by dimension reduction; N^DR^: revised N (node) stage by dimension reduction; Grade^DR^, revised tumor grade by dimension reduction; ER^DR^, revised estrogen receptor status by dimension reduction.
