# Supplementary material for: Clonal evolution characteristics and reduced dimension prognostic model for non-metastatic metachronous bilateral breast cancer
Source: Front Oncol. 2022 Sep 29;12:963884. doi: 10.3389/fonc.2022.963884 (PMC9559188; doi:10.3389/fonc.2022.963884)
Supplement: Supplementary file 5 [file DataSheet_1.pdf]

Supplementary Material

1 Supplementary Data

To obtain clean data, terminal adaptor sequences and low-quality reads (reads with a high N rate or with low-quality bases) were removed from the raw data. The clean reads were aligned to human genome build GRCh37 using Burrows-Wheel Aligner (BWA, http://bio-bwa.sourceforge.net) with default parameters. GATK and MuTect2 (https://gatk.broadinstitute.org/hc/en-us/articles/360037593851-Mutect2) were used to call somatic single-nucleotide variations (SNVs) and somatic small insertions and deletions (Indels). SNVs and Indels with the variant allele fraction (VAF) ≥2% were retained. ANNOVAR software was used to annotate the candidate mutations to genes to identify the mutated protein-coding position and exclude intronic and silent changes. GATK(https://gatkforums.broadinstitute.org/gatk/discussion/9143/how-to-call-somatic-copy-number-variants-) were used detect copy number variants (CNVs). Finally, all the candidate variants were verified with a manual visual inspection in the integrative genomics viewer browser to further remove artifactual changes.

2 Protocol for the paraffin sections of postoperative specimens from patients with metachronous or synchronous bilateral breast cancer

The population:

The patients with metachronous or synchronous bilateral breast cancer without metastatic lesions at diagnosis from the First Hospital of Jilin University during 2001-2019

Inclusion criteria:

1. The patient was not less than 18 years at diagnosis. 2. The patient was re-diagnosed with contralateral primary breast cancer after the initial diagnosis of breast cancer regardless of the interval diagnosis time. 3. According to the Third Edition of the International Classification of Diseases for Oncology (ICD-O-3), the both lateral primary tumor lesions were malignant. 4. The follow-up time was more than or equal to 3 months.

Exclusion criteria:

1. The patient had distant metastasis. 2. The follow-up information was lost. 3. The male patients.

Estimated patient sample size:

100 paraffin postoperative specimens from 50 bilateral breast cancer patients (2 specimens from bilateral lesions for each patient)

Collection and preservation for patient samples and information:

Paraffin specimens of breast cancer after operation were stored in the Specimen Bank of Pathology Department of the First Hospital of Jilin University. Paraffin specimens of cancer tissues or adjacent tissues of each lateral lesion of each patient were sectioned, and each paraffin block had 10 slices with a thickness of 6um, which were used for DNA whole-exome sequencing. The following information was collected for each patients: age at diagnosis, time interval between diagonosis of bilateral lesions, race, marital status, family history, complication, pathological type, grade, tumor size lymph nodes metastasis, estrogen receptor status, progesterone receptor status, human epidermal growth factor receptor 2 status, surgery method, follow-up time and outcomes including living at the end of the follow-up, death from cancer, and death from non-cancer causes.

3 Statement:

The protocol was in accordance with the precepts of the Helsinki Declaration and approved by the Ethics Committee of the First Hospital of Jilin University. The informed consents were obtained from each participant or each participant's guardian.
